# P-1853. Is it worth the shot? Costs associated with Long acting Dalbavancin use compared to Standard of Care Antibiotics at VA North East Ohio Healthcare System

**DOI:** 10.1093/ofid/ofaf695.2022

**Published:** 2026-01-11

**Authors:** Dale Zachary Gozum, Robbie Lee Anne Christian, Brigid Wilson, Jessica Banks, Federico Perez, Lewis S Musoke

**Affiliations:** Case Western Reserve University, University Hospitals, Cleveland, OH; VANEOHS , Cleveland, OH; VA Northeast Ohio Healthcare System, Cleveland, Ohio; VA NorthEast Ohio Healthcare System, Cleveland, Ohio; Louis Stokes Cleveland VA, Cleveland Heights, Ohio; Veterans Affairs Northeast Ohio Healthcare System, Cleveland, OH

## Abstract

**Background:**

Long acting Dalbavancin, a semisynthetic glycopeptide has been demonstrated to be safe and effective in the management complicated gram positive infections.

Due to prohibitive costs, Dalbavancin is used sparingly compared to standard of care antimicrobial agents. VA NorthEast Ohio Healthcare System (VANEOHS) OPAT program outsources their services to private infusion and nursing agencies which can often vary in costs. Dalbavancin’ s long half-life provides the potential to forego several operational costs associated with the administration of standard of care agents in the ambulatory care setting.

VANEOHS has a well-established OPAT program that has been using Dalbavancin since 2014 demonstrating similar trends in efficacy and safety as noted by Dr. Christian et. al 2025.

We sought to compare the direct medical cost to the medical center of using IV Dalbavancin, compared to standard of care antimicrobial agents for OPAT.

**Methods:**

A single center retrospective review of patients receiving OPAT at VANEOHS from fiscal year (FY) 2022 to 2025.

We queried both VHA pharmacy and community claims databases to compare cost of Dalbavancin compared to SoC agents as our primary outcome.

We excluded patients receiving OPAT in acute care settings, skilled nursing facilities and dialysis centers. Patient also receiving multiple IV antimicrobial agents for OPAT were excluded.

**Results:**

A total of 73 courses of Dalbavancin were included in the study. 60% of the Dalbavancin courses were single dosed and 40% of the Dalbavancin courses were 2 or more doses. The total cost of claims paid for Dalbavancin across the study years was $250,000 compared to $271,000 in SoC. Claims for single dalbavancin doses cost $118,363.71 compared to $110,705 in SoC. Claims for 2 or more doses of Dalbavancin cost $153,098.93 compared to SoC $133,258.

**Conclusion:**

Our study demonstrates that receiving one or multiple of IV Dalbavancin is more costly than the standard of care options. However, other metrics such as length of stay, PICC line associated complications and weekly health care encounters continue to make Dalbavancin an appealing option. Further studies are need to evaluate the cost effectiveness of Dalbavancin as an agent.

**Disclosures:**

All Authors: No reported disclosures

